# Two cases of radiation-associated angiosarcoma of the breast

**DOI:** 10.1186/s40792-018-0539-8

**Published:** 2018-11-13

**Authors:** Yuki Nomoto, Yuko Kijima, Yoshiaki Shinden, Munetugu Hirata, Yuka Eguchi, Heiji Yoshinaka, Ikumi Kitazono, Tsubasa Hiraki, Akihide Tanimoto, Shoji Natsugoe

**Affiliations:** 1 Department of Digestive Surgery, Breast and Thyroid Surgery, Kagoshima University Hospital, 37-1 Uearata, Kagoshima, 890-0055 Japan; 2 Department of Breast Surgery, Kagoshima City Hospital, Kagoshima, Japan; 3 Department of Pathology, University Graduate School of Medical and Dental Sciences, Kagoshima, Japan

**Keywords:** Angiosarcoma, Radiation therapy, Breast-conserving treatment, Secondary angiosarcoma, Breast cancer

## Abstract

**Background:**

The incidence of radiation-associated angiosarcoma (RAA) of the breast has been increasing, and its prognosis is reportedly poor. It is important to remove tumor tissues completely to prevent recurrence.

**Case presentation:**

We report two cases of patients with RAA of the breast. Both patients had a nodule in their remaining breast a few years after undergoing breast-conserving surgery and radiation therapy for breast cancer. The nodules were diagnosed as angiosarcoma by skin biopsy and open biopsy, respectively. To determine the extent of lesion spread, mapping biopsy was performed before surgery. Both patients underwent mastectomy, extensive skin resection, and split skin grafting. Pathological findings showed that their tumors could be completely resected. After surgery, chemotherapy was performed.

**Conclusion:**

In our cases, no local or distant recurrence has been detected in either patient for over 4 years. We identified the range of tumor invasion by preoperative mapping biopsy and completely resected all tumor tissue.

## Introduction

Radiation-associated angiosarcoma (RAA) of the breast is an unpleasant complication of radiation therapy for breast cancer. In the last few decades, breast-conserving surgery (BCS) with radiation therapy has replaced mastectomy as the standard of care for early-stage breast cancer [1]. Consequently, the incidence of RAA of the breast has been increasing, with the cumulative incidence reported to be 0.9 per 1,000 breast cancer cases in the past 15 years [2]. The prognosis of patients with RAA is reportedly poor. The recommended treatment for RAA of the breast is surgical resection. Although several reports about RAA of the breast have been published, methods for determining the resection range are not clearly described in these reports.

Here, we report two cases in which we identified the range of tumor invasion by preoperative mapping biopsy and resected the tumor tissue completely.

## Case report

### Case 1

**Fig. 1:**
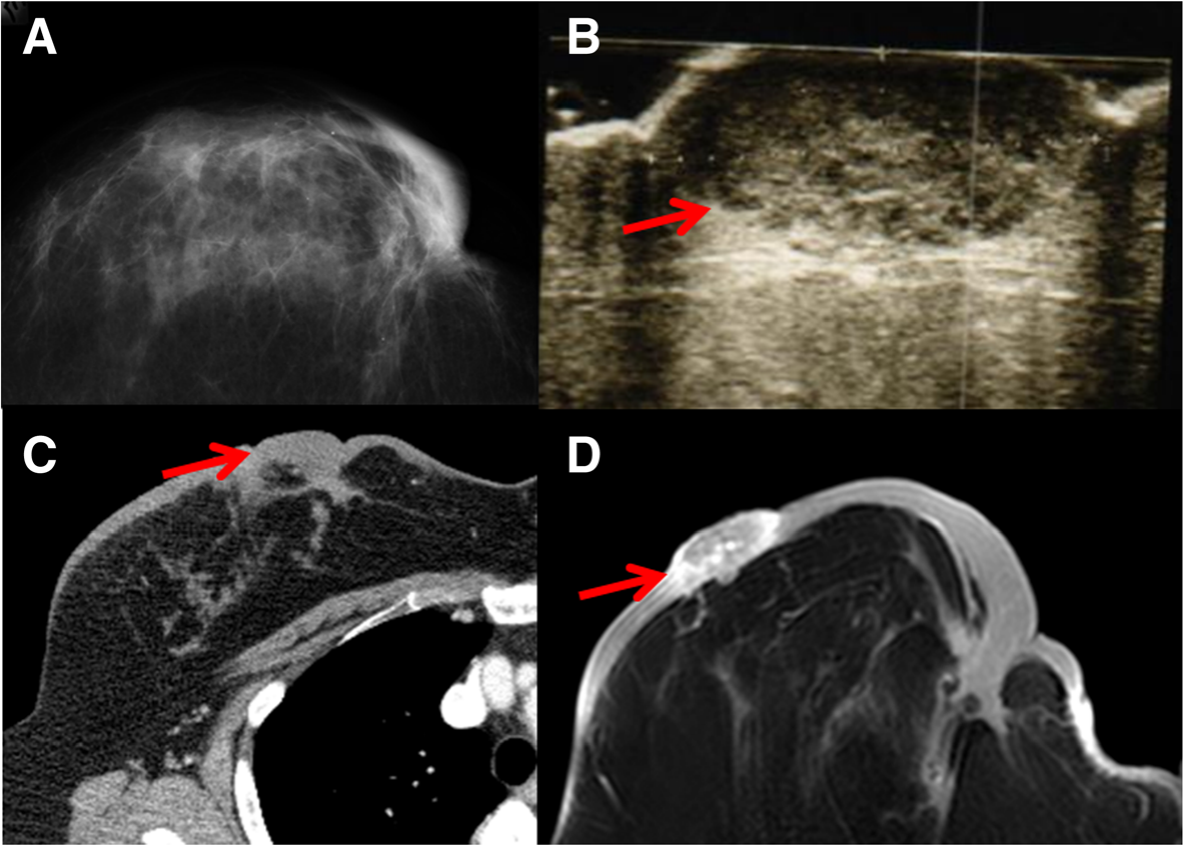
Imaging findings (case 1). **a** Mammography. **b** Breast ultrasound. **c** Computed tomography. **d** Magnetic resonance imaging. Arrow: tumor

**Fig. 2:**
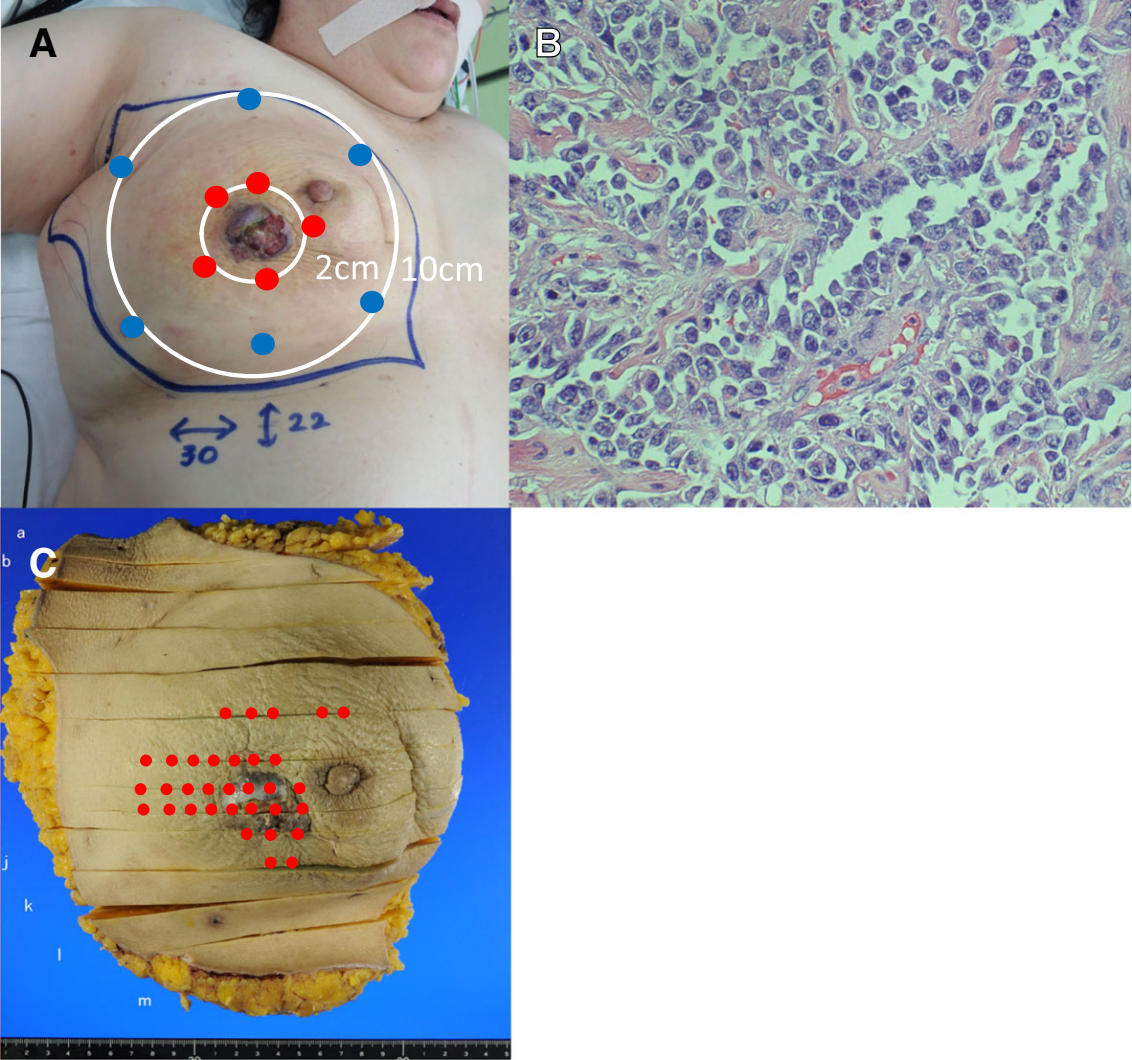
Macroscopic and pathological findings (case 1). **a** Mapping biopsy was performed at 2 cm and 10 cm from the nodule. Red dots indicate positivity for tumor cells, and blue dots indicate negativity. **b** Pathological image of the resected specimen. It was diagnosed as angiosarcoma (hematoxylin-eosin [H.E.] staining: × 400). **c** Red dots indicate where tumor cells were observed. Tumor invasion was observed in a wider area outside the nodule

A 64-year-old woman visited our hospital with a 1-month history of a 1-cm dark red nodule in her right breast. Four years before, she underwent BCS and axillary lymph node dissection for right breast cancer followed by endocrine therapy and radiation therapy. The nodule was diagnosed as angiosarcoma by skin biopsy. A variety of image examination revealed a mass of 27 × 13 mm in outer lower lesion of her right breast, and the surrounding skin was markedly thickened (Fig. 1). Mapping biopsy 2 cm from the edge of the nodule revealed tumor invasion in all five sites examined, while mapping biopsy at 5 cm or 10 cm revealed no tumor invasion in any of the six sites examined (Fig. 2a).

Total mastectomy with extensive skin resection (30 × 22 cm) was performed. The resection line was 10 cm from the edge of tumor. To repair a large skin defect, a wide skin graft using abdominal skin was performed. The pathological diagnosis was angiosarcoma, 45 × 40 × 20 mm in size (Fig. 2b, c). The surgical margins were completely free from tumor cells. Postoperative chemotherapy (weekly paclitaxel, 80 mg/m^2^ × 6 cycles) was administered, and the patient has experienced no recurrence for 6 years, 3 months.

### Case 2

**Fig. 3:**
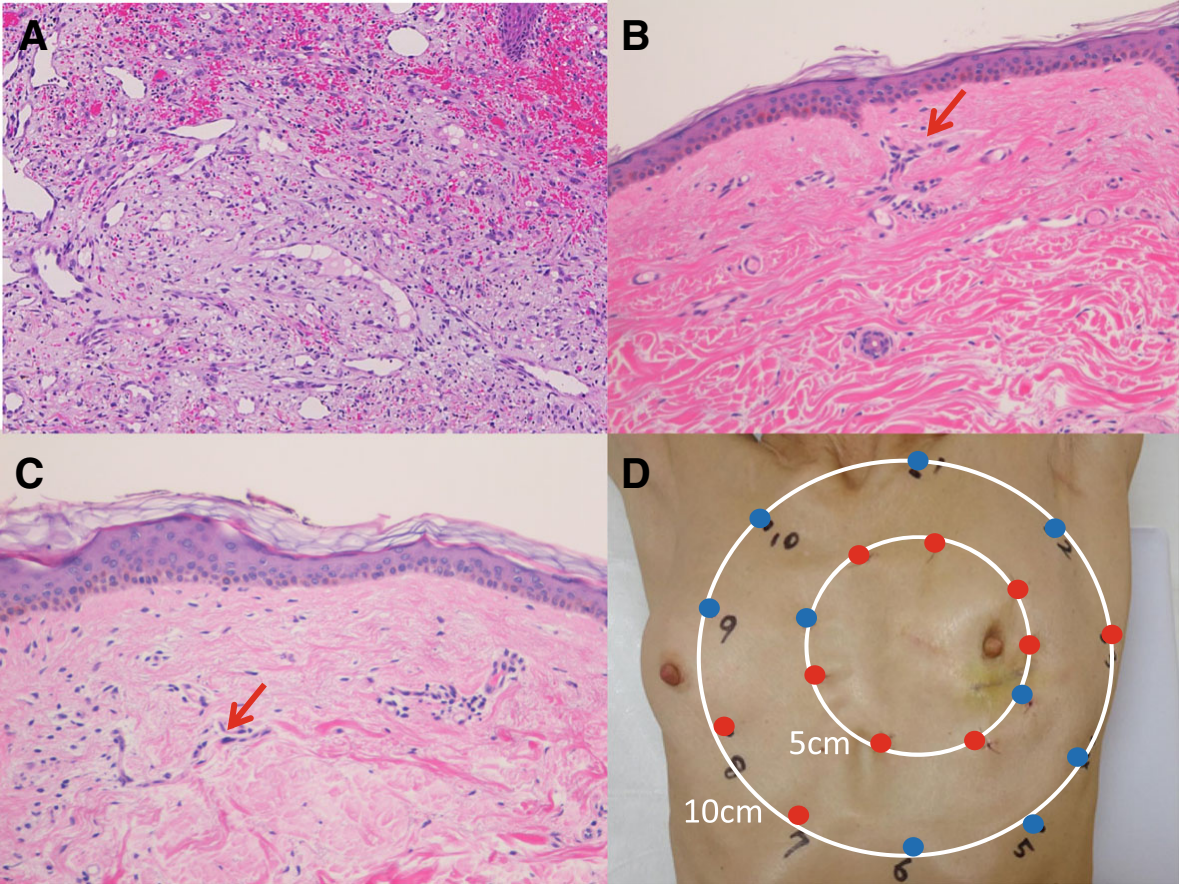
Macroscopic and microscopic pathological findings (case 2). **a** The nodule was diagnosed as angiosarcoma by open biopsy (H.E. staining: × 20). **b** Mapping biopsy 5 cm from the surgical trace (H.E. staining: × 20). Arrow: atypical endothelial cells. **c** Mapping biopsy 10 cm from the surgical trace (H.E. staining: × 20). Arrow: atypical endothelial cells. **d** Mapping biopsy was performed 5 cm and 10 cm away from the surgical trace. The red dots indicate areas where atypical endothelial cells were observed, and blue dots indicate areas where no atypical endothelial cells were observed

A 67-year-old woman had undergone BCS and sentinel lymph node biopsy for left breast cancer followed by chemotherapy, anti-HER2 therapy, and radiation therapy 3 years before. She visited another hospital with a 3-month history of a dark red nodule in her left breast. The nodule had been diagnosed as angiosarcoma by open biopsy by a dermatologist (Fig. 3a). Immunohistochemistry such as CD31 and CD34 were positive. She then consulted our department for surgical treatment. We could not point out obvious abnormal findings in imaging findings.

Seven out of nine points of a mapping biopsy 5 cm from the surgical trace revealed atypical endothelial cells (Fig. 3b), while three out of ten points of mapping biopsy at 10 cm revealed atypical endothelial cells (Fig. 3c).

She underwent left mastectomy with extensive skin resection (25 × 20 cm). The resection range exceeded the three sites at which atypical endothelial cells were observed, and in other places, a range of 10 cm from the surgical trace was used. To repair a large skin defect, a wide skin graft from the thigh was performed.

**Fig. 4:**
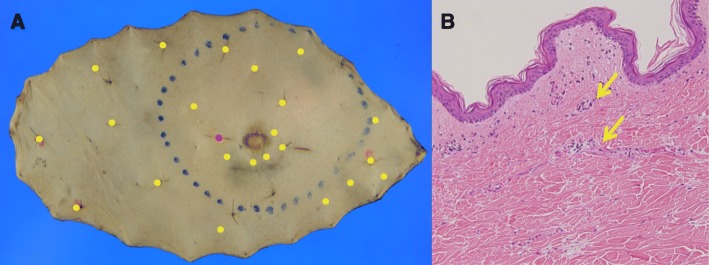
Macroscopic findings of mapping biopsy and pathological examination (case 2). **a** A red dot indicates the surgical trace. Yellow dots indicate locations at which atypical endothelial cells were observed. **b** Atypical endothelial cells, in which the degree of atypia was less than that of the primary tumor (H.E. staining: × 20). Arrow: atypical endothelial cells

Atypical endothelial cells were observed in resected specimens, but the degree of atypia was less than that of the primary tumor. Atypical endothelial cells were not observed in the resection margin (Fig. 4).

Postoperative chemotherapy (nab-paclitaxel, 260 mg/m^2^ × 4 cycles) was administered, and the patient has experienced no recurrence for 5 years.

## Discussion

The mean latency period of RAA of the breast after radiation therapy is approximately 5–7 years [3]. Clinically, it presents as violaceous, erythematous plaques; nodes; areas of ecchymosis; and skin thickening [4, 5].

Cahan et al. and Arlen et al. proposed the following criteria for diagnosis of RAA of the breast: (1) a sarcoma arising within the field of previous radiotherapy, (2) differing histology between the secondary sarcoma and primary tumor, and (3) at least a 3-year latency period between radiation therapy and development of the sarcoma [6, 7]. The two present cases fulfilled all of those criteria.

Standard therapy for RAA of the breast is simple mastectomy and/or wide local excision [8], because disease-free survival is significantly shortened if the resected stump is positive for cancer cells [9]. Seinen et al. reported that the cause of high recurrence is due to multifocal growth of angiosarcoma and residual tumor tissue. These investigators reported that even if the surgical margins are considered to be clear, it is preferable to resect all irradiated skin [10]. The suggested treatment is aggressive surgery with removal of the pectoral muscle and subsequent reconstruction to achieve clear margins [11]. For deciding the area of resection, mapping biopsy is useful. Mapping biopsy is multiple skin biopsy at a certain distance from the tumor for detecting tumor invasion. By this method, it is possible to confirm the range of tumor spreading. In case 1, we confirmed the range free from invasion of tumor cells by mapping biopsy, and we resected skin with a 10-cm margin. In case 2, atypical endothelial cells were confirmed both inside and outside of the irradiation range. Atypical post-radiation vascular lesions (AVLs) have been described to arise within previously irradiated skin [12]. Therefore, we considered the possibility of tumor invasion rather than AVLs. Although skin transplantation was required, we were able to resect the tumor completely. Both case 1 and case 2 omitted the axillary operation because there was no metastasis to the axillary lymph node in the preoperative image examination.

Regarding radiation therapy for angiosarcoma, Depla suggested that the addition of reirradiation to surgery may help in local control of RAA [13]. On the other hand, Torres suggested that the use of radiation therapy remains controversial, as repeat radiation exposure to an area that has already been irradiated may result in toxicity [14]. Therefore, we did not perform postoperative radiation therapy for RAA.

Regarding chemotherapy for angiosarcoma, Sinnamon performed retrospective analysis of cutaneous angiosarcoma, and the analysis indicated both adjuvant and neoadjuvant therapy after surgery did not show any survival benefit on univariate and multivariate [15]. On the other hand, there are several reports showing the possibility that taxanes are useful for angiosarcoma [16, 17].

Angiosarcomas express VEGFR [18]. Several studies using anti-VEGF monoclonal antibody have shown antitumor activity in angiosarcoma [19, 20]. On the basis of this background, Ray-Coquard conducted a non-comparative, open-label, randomized phase 2 trial to explore the activity and safety of bevacizumab and paclitaxel therapy for patients with advanced angiosarcoma. Fifty patients were randomized and assigned to two arms: (1) the paclitaxel alone or (2) the paclitaxel and bevacizumab arm. From the findings, they concluded that there is no benefit from adding bevacizumab to paclitaxel [21].

Therefore, it is thought that chemotherapy/molecular target treatment for angiosarcoma has not yet been determined. We used a taxane for two patients, taking into account the fact that the taxane may be effective and that they did not use anthracycline after the initial breast cancer surgery.

In general, the prognosis of patients with RAA of the breast is poor. The 5-year local recurrence-free survival rate is 41–47%, and the 5-year overall survival rate is 10–54% [14, 22, 23]. The median time to local recurrence after diagnosis has been reported to be 6 months (range, 1–89 months) [10, 24]. Poor prognosis is reported to be associated with large tumor size, high histologic grade, and positive surgical margins [25].

To the best of our knowledge, several case reports of RAA in the breast have been published since 1990. In those reports, mastectomy and/or wide excision were conducted; however, no reports have clearly described a method for determination of the surgical margin.

We identified the range of tumor invasion by preoperative mapping biopsy. This technique could potentially lead to complete resection of tumor tissues and a good prognosis.

## Conclusion

Mapping biopsies are useful for confirming the invasion range of RAA of the breast. If tumor cells are completely resected, a long-term good prognosis can be achieved.
